# Association Between Living Arrangement and Acute Care Use in Older Medicare Home Health Patients

**DOI:** 10.1093/geroni/igaa057.1674

**Published:** 2020-12-16

**Authors:** Jinjiao Wang, Meiling Ying, Xueya Cai, Thomas Caprio, Helena Temkin-Greener, Yue Li

**Affiliations:** University of Rochester, Rochester, New York, United States

## Abstract

This secondary analysis used a 10% random sample from the national Outcome and Assessment Information Set (OASIS) of Medicare beneficiaries ≥ 65 years old who received home health (HH) care in 2017 (N=646,109). We examined the risk of hospital admission during a 60-day HH episode among Medicare home health patients in different living arrangements, including living alone at home (23.8%), living with other at home (64.8%), and residing in assisted facility (AL) facilities (11.4%). At the start of the HH episode, AL residents were older, more likely to have cognitive impairment, depressive symptoms, and limitations in activities of daily living (ADL) than those living at home at home (alone/with others). In the multivariable logistic regression model of hospital admission adjusting for demographic status (age, sex, race/ethnicity, Medicaid status), cognitive impairment, depressive symptoms, and ADL limitations, when compared to HH patients living with others at home (reference), AL residents were 15% less likely to have hospital admission (Odds Ratio [OR]=0.85, 95% Confidence Interval [CI]: 0.84, 0.88, p<0.001). HH patients living at home alone were not statistically significantly different from the reference (OR=0.99, 95% CI: 0.98, 1.01, p=0.47). HH patients in AL, despite having worse cognitive, mental, and physical function at baseline, had better outcomes than those living at home. This suggests 1) older adults living at home may have unmet health or personal care needs, and 2) synergies may exist between post-acute care through HH care and long-term care and support at AL that are critical to patient welfare.

